# Cancellation of elective cases in pediatric surgery: An audit

**DOI:** 10.4103/0971-9261.71748

**Published:** 2010

**Authors:** Sapna Bathla, Anup Mohta, Aikta Gupta, Geeta Kamal

**Affiliations:** Department of Anaesthesiology, Chacha Nehru Bal Chikitsalaya, Affiliated to Maulana Azad Medical College, Geeta Colony, Delhi - 110 031, India; 1 Department of Pediatric Surgery, Chacha Nehru Bal Chikitsalaya, Affiliated to Maulana Azad Medical College, Geeta Colony, Delhi - 110 031, India

**Keywords:** Elective surgery, operating room, pediatric

## Abstract

**Aim:**

To determine the main reasons for cancellation of elective cases on scheduled date of surgery in pediatric patients.

**Materials and Methods:**

The audit was conducted in a 216 beds tertiary care pediatric super-specialty hospital. Two operation theatres (OT) provide elective surgical services to pediatric surgery, orthopedics, ophthalmology and otorhinolaryngology. The audit included all those patients who were posted for elective surgery over a period of one year. Cancelled cases were identified from predesigned OT utilization formats and the reasons for cancellation were evaluated.

**Results:**

A total of 2473 cases were posted for the elective surgery in the year 2009 and 189 (7.64%) patients had their surgery cancelled. The main reasons for cancellation were upper respiratory infections (30.68%) and shortage of time (29%). Other reasons were medically unfit patients (15.34%), precedence of emergency cases (3.7%); non-availability of ventilator and intensive care bed (4.7%); no-show by patient (4.76%); non-availability of blood (4.2%); incomplete work up (2.64%); administrative reasons (1.58%); patient not fasting (1.58%) and unspecified reasons (2.1%). Overall, 38.6% causes were preventable.

**Conclusions:**

Elective surgery cancellation is a significant problem with multifactorial etiology. Most common reasons for cancellation of planned surgery were sudden onset of respiratory tract infection in the admitted patient and shortage of time. It suggests that on many occasions, surgeons take more time than anticipated for performing the procedure.

## INTRODUCTION

Cancellation of surgery puts undue pressure on hospitals in view of increase in hospital expenses.[1,2] Cancellation of an elective surgery increases the patient’s stay in the hospital and associated inconvenience. It leads to waste of time for the surgeon and other support staff as well as underutilization of operation theatre. Therefore cancellation of cases on the day of surgery are generally not desirable, especially in a pediatric set up where family member(s) of the patients may miss more workdays when surgery is cancelled.[3] Pediatric patients are likely to fall ill more easily and are more vulnerable for postponement of their surgery.

Cancellation of surgery is one of the undesired events that needs to be monitored routinely in the hospital as it has adverse implications on health system and patients.

## MATERIALS AND METHODS

This retrospective observational study was conducted at a public sector tertiary care pediatric hospital. The period of the audit was from January to December 2009. This audit was conducted to (a) review the reasons for cancellation of surgeries in pediatric set up and (b) suggest ways to decrease cancellations. The main theatre complex has two theatres. Both the theatres are available from 9 AM to 4 PM from Monday to Friday whereas from 9 AM to 1 PM on Saturday. Last case under general anesthesia is taken by 3 PM. The operation theatre list is submitted on or before 2 PM the previous day. Any addition to the list is allowed till 4 PM. Cancellation was defined as the patients name being on the published OR list but did not undergo surgery on the day scheduled. The cancellation rate was calculated from the OT formats at the end of the month and reasons for cancellation were mentioned in OT cancellation spectrum.

A pre-devised operation theatre proforma is used to record patients’ demographic profile along with starting time, ending time, and actual duration of the procedure. Reasons for cancellation are also recorded. Scheduling and booking of cases is done by surgeons a day prior and a list with patients name, diagnosis, proposed procedure and anticipated surgical time is submitted. Surgery days and surgical units are fixed for different specialties. Cases are performed serially as per list allowing 10 minute gap between two cases for OT sterilization practices.

## RESULTS

A total of 2473 cases were posted for the elective surgery in the year 2009 and out of this, 189 patients had their surgery cancelled (7.64%) for various reasons. Reasons for cancellations are summarized in Table 1. Two main reasons were upper respiratory tract infections (URTI) and shortage of operating time. Out of these, reasons at 1, 3–6 were considered non-preventable (59.3%) while 2, 7–10 (39%) were considered preventable.

**Table 1:** Reasons of cancellation of elective surgery

Reason for cancellation	Frequency *N*=189 (%)
Upper respiratory tract infection	58 (30.7)
Shortage of operating time	55 (29)
Medically unfit patients	29 (15.3)
Precedence of emergency cases	7 (3.7)
Non-availability of ventilator and intensive care bed	9 (4.8)
No-show by patient	9 (4.8)
Non-availability of blood	8 (4.2)
Incomplete work up	5 (2.6)
Administrative reasons	2 (1.1)
Patient not fasting	3 (1.6)
Unspecified reasons	4 (2.1)

## DISCUSSION

Anticipation of surgery is stressful both for patients and families especially if the patient is a child. Cancellation of surgery on the scheduled date imposes further stress and anxiety. Therefore the issue of cancellation is of prime concern and every sincere effort must be taken to prevent or minimize these. Cancellation results in extremely negative feelings in some patients while others conceal their sadness.[4] Moreover it is very difficult to keep a child fasting for long and is unethical to cancel the case after prolonged fasting. Cancellation of surgical procedures leads to wastage of hospital resources and monetary as well as psychological trauma to the patients and their families.

Many retrospective and prospective studies have revealed various reasons for cancellations and have reported the rate of cancellation from 10 to 40%.[5] Haana *et al*.[6] from Australia have investigated case cancellations on the intended day of surgery at a pediatric hospital and reported cancellation rate of 7.2% of all scheduled operations. This study found medically unfit patient; operation found not necessary; postponement due to patient condition; and patient failure to attend/late as the four most common reasons for cancellations. Most common reason for cancellation on the day of surgery was URTI. This is one of the most controversial issues in pediatric anesthesia.[7] Although a child with URTI is a challenge to anesthesiologist, opinion is divided on whether to take up a patient with URTI for elective surgery. Many studies support that children with URTI should get their surgery postponed till they are asymptomatic.[8,9] Unfortunately there is no consensus on the optimal time to wait before surgery is rescheduled.[10–13] Although for certain population there appears to be no increased risk and case can be managed with minimum morbidity.[14,15] The literature supports selective cancellation of surgery for these children.[16] So decision should be on case to case basis and blanket cancellation of child with URTI should not be done.

Cancellation rate of these children with URTI was high (30.7%) in the present study when compared to data found in a national survey.[17] Another common reason for cancellation was due to list overrun (short of time). The surgeons took longer than the anticipated surgical time leading to cancellation of cases posted later in the list. This was more common with few surgeons who repeatedly underestimated the expected time and hence cancellation rate was much higher with them. Similar finding has been reported by Schofield *et al*.[18] as one of the most common reason for cancellation of surgery. This is something which is avoidable and the O.T. list should be scheduled by the chief surgeon of the next day in consultation with the other operating surgeons so that the concerned person can calculate his expected time according to his requirement. It is important as different surgeons may take different time for same procedure. Also it must be considered that sometimes surgeon may encounter different than anticipated or complex surgical findings necessitating change of surgical procedure leading to longer surgical time. Improved methods of booking and allocating theatre time and operating room technique for patient flow can be used for quality improvement. Operation lists should be prepared to avoid over or under-utilization.

In order to avoid posting of medically unfit patients in the elective list, good preoperative evaluation is required to be done by an experienced anesthesiologist. So it is recommended that the anesthesiologist responsible for the case in theatre should do the preoperative evaluation.[19] Due to theatre time constraints, sometimes surgeons used their discretion to accommodate a semi-emergency surgery like malignancy after deferring a planned case. Such cases take a priority over elective cases but disrupt an elective list. Cancellation due to non-availability of intensive care bed and ventilator required cases was also noted.

Sometimes patients do not turn up or deny consent for surgery at the last minute leading to cancellation. Most surgical centers in the West levy fee for cancellation of elective surgery.[20,21] However, we can reduce this by proper communication with the parents. On many occasions, the investigations recommended by anesthesiologists are not done leading to cancellation. Sometimes parents may give feed to the child in the morning of surgery despite clear instructions by an anesthesiologist and the nursing personnel. This may also happen if the caregiver who has been given instructions is replaced by another one in the night which is quite common in our circumstances. Non-availability of blood for major surgery is an avoidable cause of cancellation. Surgeons should ensure that adequate necessary blood products suggested by an anesthesiologist in-charge are arranged. Sometime it may become necessary to release the reserved blood for some other patient as a life-saving measure resulting in non-availability of blood or blood products for an elective case posted next morning. Sometimes cancellation may become necessary due to administrative/logistic reasons beyond control.

To conclude, at times cancellation of surgery becomes necessary as it happens in our circumstances due to non-preventable reasons like medical illness, but one should aim to prevent or minimize cancellation by careful planning and making protocols to use the resources optimally and avoid financial loss and psychological trauma to the parents and patients.

## References

[1] Boothe P, Finegan BA (1995). Changing the admission process for elective surgery: An economic analysis. Can J Anaesth.

[2] Rai MR, Pandit JJ (2003). Day of surgery cancellations after nurse led pre-assessment in an elective surgery centre: The first 2 years. Anaesthesia.

[3] Tait AR, Voepel-Lewis T, Munro HM, Gutstein HB, Reynolds PI (1997). Cancellation of paediatric out-patient surgery: Economic and emotional implications for patients and their families. J Clin Anesth.

[4] Sevgi D, Fatma E (2004). The causes and consequences of cancellations in planned orthopedic surgery: The reactions of the patients and their families. J Ortho Nurs.

[5] Garg R, Bhalotra AR, Bhadoria P, Gupta N, Anand R (2009). Reasons for cancellation of cases on the day of surgery –A Prospective Study. Indian J Anaesth.

[6] Haana V, Sethuraman K, Stephens L, Rosen H, Meara JG (2009). Case cancellations on the day of surgery: An investigation in an Australian paediatric hospital. ANZ J Surg.

[7] Tait AR, Malviya S (2005). Anaesthesia for the child with an upper respiratory tract infection: Still a dilemma?. Anesth Analg.

[8] Cohen MM, Cameron CB (1991). Should you cancel the operation which a child has an upper respiratory tract infection?. Anesth Analg.

[9] Parnis SJ, Barker DS, Van Der Walt JH (2001). Clinical predictors of anaesthetic complications in children with respiratory tract infections. Paediatr Anaesth.

[10] Tait AR, Malviya S, Voepel-Lewis T, Munro HM, Seiwert M, Pandit UA (2001). Risk factors for perioperative adverse respiratory events in children with upper respiratory tract infections. Anesthesiology.

[11] Empey DW, Laitinen LA, Jacobs L, Gold WL, Nadle JA (1976). Mechanisms of bronchial hyperreactivity in normal subjects after upper respiratory tract infection. Am Rev Respir Dis.

[12] Aquilina AT, Hall WJ, Douglas RG, Utell MJ (1980). Airway reactivity in subjects with viral upper respiratory tract infections: The effects of exercise and cold air. Am Rev Respir Dis.

[13] Tait AR, Reynolds PI, Gutstein HB (1995). Factors that influence an anesthesiologists decision to cancel elective surgery for the child with an upper respiratory tract infection. J Clin Anesth.

[14] Berry FA (1984). Preexisting medical conditions of pediatric patients. Semin Anesth.

[15] Tait AR, Knight PR (1987). The effects of general anaesthesia on upper respiratory tract infections in children. Anaesthesiology.

[16] Elwood T, Morris W, Martin L, Nespeca MK, Wilson DA, Fleisher EA (2003). Bronchodilator premedication does not decrease respiratory adverse events in pediatric general anesthesia. Can J Anaesth.

[17] Cote CJ (2001). The upper respiratory tract infection dilemma: Fear of complication or litigation?. Anaesthesiology.

[18] Schofield WN, Rubin GL, Piza M, Lai YY, Sindhusake D, Fearnside MR (2005). Cancellation of operations on day of intended surgery at a Major Australian referral hospital. Med J Aust.

[19] Down MP, Wong DT, McGuire GP (1998). The anaesthesia consult clinic: Does it matter which anaesthetist sees the patient?. Can J Anaesth.

[20] Coastal Health Centre, Ellsworth ME, USA. Patient information sheet. http://www.mcmhospital.org/departments/patient-info-drn.pdf.

[21] American Medical Association. Code of ethics. Appointments changes. http://www.ama-assn.org/ama/pub/category/8466.html.

